# Correction to “Outcomes of patients with relapsed or refractory primary mediastinal B‐cell lymphoma treated with anti‐CD19 CAR‐T cells: CARTHYM, a study from the French national DESCAR‐T registry”

**DOI:** 10.1002/hem3.70335

**Published:** 2026-03-25

Galtier J, Mesguich C, Sesques P, et al. Outcomes of patients with relapsed or refractory primary mediastinal B‐cell lymphoma treated with anti‐CD19 CAR‐T cells: CARTHYM, a study from the French national DESCAR‐T registry. *HemaSphere*. 2025;9(2):e70091. doi:10.1002/hem3.70091

In Table 1, the ranges for the ages at first infusion were missing. These have now been added.

Additionally, a typographical error in the last sentence of the Abstract has been corrected; this sentence now reads as “In conclusion, this study confirms that anti‐CD19 CAR‐T cells is a valid standard‐of‐care…”.

The original article has been updated. We apologize for this error.

